# Fluorescent labeling of human albumin using the new aromatic dialdehyde labels and the study of innerfilter effect

**DOI:** 10.4103/0975-7406.72143

**Published:** 2010

**Authors:** Muhammad Aminuddin, Sofia Ahmed, Muhammd Ali Sheraz, Iqbal Ahmad, Karamat Mahmood, J. N. Miller

**Affiliations:** Institute of Pharmaceutical Sciences, Baqai Medical University, Toll Plaza, Super Highway, Gadap Road, Karachi - 746 00, Pakistan; 1 Department of Chemistry, The Islamia University, Bahawalpur, Pakistan; 2 Department of Analytical Chemistry, Loughborough University of Engineering & Technology, Loughborough, LE11, England

**Keywords:** Fluorescent labeling, fluorophores, innerfilter effect, quenching

## Abstract

The labels naphthalene-2,3-dicarboxaldehyde (NDA), 1-phenylnaphthalene-2,3-dialdehyde (ΦNDA), and anthracene-2,3-dialdehyde (ADA) have been used as fluorigenic reagents. They formed fluorescent derivatives with proteins. The derivatives formed are in fact isoindoles. The fluorescence decay of the labels-antibody was found to extend over a period of 4, 8, and 10 h for ΦNDA, ADA, and NDA-derivative, respectively. Protein formed is comparatively less stable as compared to simple amino acids. In relation to innerfilter effect, the addition of cytochrome C, myoglobin, and ATP as absorbers to label-human albumin fluorophores appeared to have quenched the fluorescence. In the case of using NDA as label, the fluorescence was quenched roughly 70%, 24%, and 58% for addition of cytochrome C, myoglobin, and ATP, respectively. The labels used were found to give rapid, reproducible, and reliable results.

Proteins absorb in the region 270–300 nm and possess some degree of fluorescence mainly due to aromatic amino acid residues, tyrosine and tryptophan. However, other fluorescent structures bound to the protein other than peptide linkages may also contribute to fluorescence. Proteins can also be combined chemically with the fluorigenic reagents to yield fluorescent protein derivatives. The fluorigenic reagents may find application as an end group reagent.[1] In the present study, an effort has been made to investigate the fluorescence properties of the different dialdehydes[2] – the naphthalene-2,3-dicarboxaldehyde (NDA), 1-phenylnaphthalene-2,3-dialdehyde (ΦNDA), and anthracene-2,3-dialdehyde (ADA) protein derivatives with special reference to their stability and ease of formation along with the innerfilter effect (IFE). The dialdehyde–protein (antibody) derivative appears to be highly fluorescent.

The above noted fluorigenic reagents form fluorescent isoindoles. Besides these, there are other fluorigenic reagents[3–8] which form fluorescent derivatives. Of these o-phthalaldehyde (OPA) is used for amino functional groups forming the isoindole product. The stability of the fluorescent derivative depends upon a number of factors; the thiol structure greatly affects the fluorescence intensity as well as the stability of the reaction product.

Absorption of excited and/or emitted radiation by dissolved species, including the fluorophore itself, is termed the IFE.[9] Measurements of fluorescence quenching are widely used in quantitative chemistry and biochemistry. However, in the filtereffect there is a decrease in fluorescence intensity, it is not a quenching effect.[9] The IFE in fluorescence spectroscopy is not easily distinguished from dynamic and static quenching phenomena. Since IFE rarely occurs without quenching, IFE correction has also been the focus for improved results.[10] This paper aims to report the application of the NDA, ΦNDA, and ADA labels for reaction with protein. Important reactions will focus on the stability of the product including the study of IFE using the fluorophores in the presence of absorbers such as cytochrome C, myoglobin, and ATP.

## Materials and Methods

### Materials

Fluorigenic reagents (labels NDA, ΦNDA, and ADA) were synthesized in the laboratory at Loughborough University of Engineering and Technology, UK. Human albumin (HAlb) economy was obtained from DAKO-immunoglobulins Ltd., USA, whereas cytochrome C, myoglobin, ATP (adenosine 5’-triphosphate disodium salt), β-cyclodextrin and methyl-2-propanethiol, and ethanethiol were obtained from Sigma Chemicals Co. Ltd., USA. All other reagents used were of analytical grade.

Fluorescence measurements were generally made either on an MPF-44 β spectrofluorimeter (Perkin-Elmer) or Baird Atomic model SFR 100.

### Procedure

A dilute solution of the sample (0.2–3.0 ml) was treated with 1 ml of 10.9 mM β–cyclodextrin (β-CD). Borax-sodium hydroxide buffer (0.6 ml, pH 10.0), 0.1 ml aqueous thiol solution (0.1%v/v) were then added, followed by the addition of suitable fluorigenic reagent in slight excess over the sample. The final volume was then made up to 5 ml with water. The derivative was then excited at a suitable wavelength for fluorescence emission measurement.

Human albumin economy (antibody) was derivatized with NDA, ΦNDA, and ADA separately and then these derivatives were used for studies.

## Results and Discussion

### Stability

**Figure 1:**
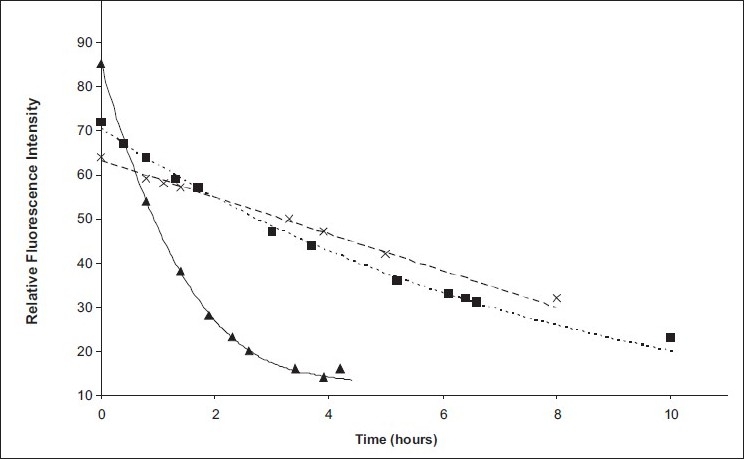
Stability curves of ΦNDA (▲), NDA (■), and ADA–antibody (×)

The NDA, ΦNDA, and ADA fluorescent protein derivatives have been studied to determine their stability. The fluorescence intensities of the derivatives were measured at regular time intervals. Figure 1 indicated that the fluorescence decay of the ΦNDA, ADA, and NDA-antibody was found to extend over a period of 4, 8, and 10, h, respectively. The stability of protein derivatives in comparison with simple amino acid derivatives was a little less; the fluorescence intensity was reduced to its minimum in less than half of the time of simple amino acid derivatives. The increase in instability may be due to the unfolding of the labeled protein leading to conformational alteration of the protein macromolecules.

### Innerfilter effects

An insignificant decrease in the fluorescence intensity of the fluorescent species may result by the addition of an absorbing molecule to it. The absorption of light by the molecule at the wavelength of emission is called secondary absorption and this IFE needs to be compensated before carrying out a quantitative analysis of the results. The present study attempts to investigate such an effect on the label–human albumin fluorophores using cytochrome C, myoglobin, and ATP as absorbers.

**Figure 2:**
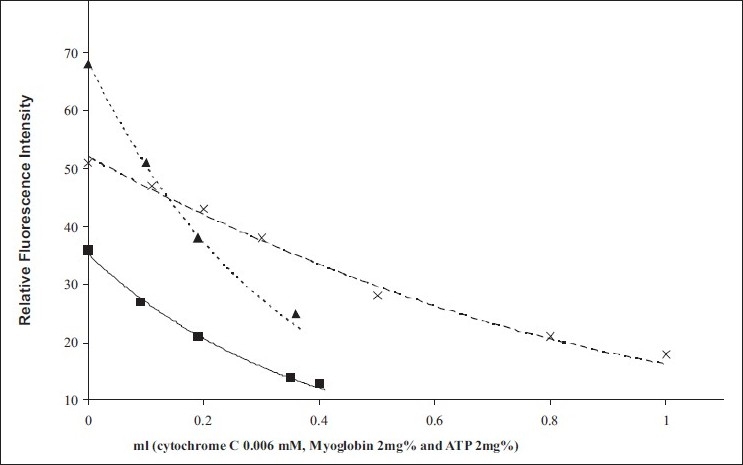
Innerfilter effect resulting from the addition of different absorbers to the NDA–human albumin (HAlb) fluorophore (different initial concentration). NDA-HAlb, purified/cytochrome C (■); NDA–HAlb, purified/myoglobin (×); NDA–HAlb, purified/ATP (▲)

In Figure 2, the IFE resulting from the addition of cytochrome C, myoglobin, and ATP to the NDA–HAlb fluorophores is shown by the three curves. These curves represented the uncorrected fluorescence which may be corrected by applying the equation of Geren and Millett.[11]

$$F_{c}\;=\;F_{o}\;\times \;{\mathrm{antilog}}_{e}\;(A_{1}\;+\;A_{2}/2)$$

where *F_c_* is the corrected fluorescence, *F_o_* is the observed fluorescence corrected for dilution, and A_1_ and A_2_ are the primary and secondary absorbance, respectively.

For an addition of 0.4 ml of the absorbers, the fluorescence was quenched roughly by 70%, 24%, and 58% for addition of cytochrome C, myoglobin, and ATP, respectively [Figure 2]. However, the IFE in the case of ΦNDA and ADA by cytochrome C was found to be small.

**Figure 3:**
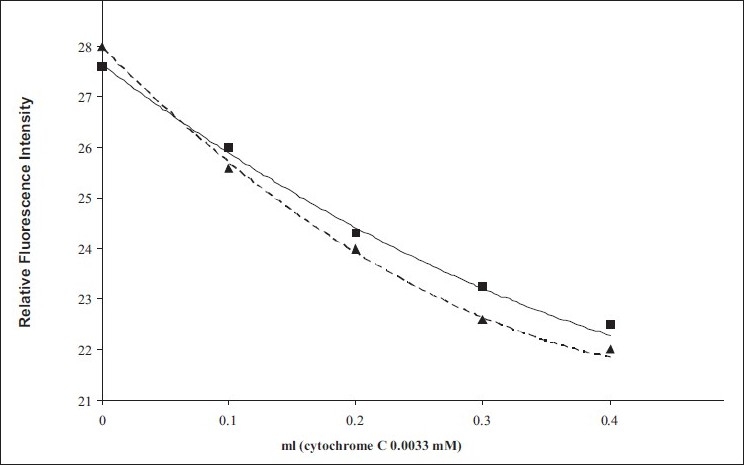
Innerfilter effects due to addition of absorber (cytochrome C) to ΦNDA (■) and ADA (▲)-human albumin fluorophores

The derivatives of HAlb formed with other fluorigenic reagents ΦNDA and ADA showed a similar quenching of fluorescence [Figure 3] by cytochrome C.

The selection of these absorbers for the study was due to the fact that all these are nonfluorescent. Cytochrome C and myoglobin are noteworthy in that in spite of being proteins they are nonfluorescent due to the quenching effect of the heme group present.[12]
